# Beta- and gamma-range human lower limb corticomuscular coherence

**DOI:** 10.3389/fnhum.2012.00258

**Published:** 2012-09-11

**Authors:** Joseph T. Gwin, Daniel P. Ferris

**Affiliations:** Human Neuromechanics Laboratory, School of Kinesiology, University of MichiganAnn Arbor, MI, USA

**Keywords:** EEG, EMG, beta band, gamma band, coherence, isometric, isotonic, muscle contraction

## Abstract

Coherence between electroencephalography (EEG) recorded on the scalp above the motor cortex and electromyography (EMG) recorded on the skin of the limbs is thought to reflect corticospinal coupling between motor cortex and muscle motor units. Beta-range (13–30 Hz) corticomuscular coherence has been extensively documented during static force output while gamma-range (31–45 Hz) coherence has been linked to dynamic force output. However, the explanation for this beta-to-gamma coherence shift remains unclear. We recorded 264-channel EEG and 8-channel lower limb EMG while eight healthy subjects performed isometric and isotonic, knee, and ankle exercises. Adaptive mixture independent component analysis (AMICA) parsed EEG into models of underlying source signals. We computed magnitude squared coherence between electrocortical source signals and EMG. Significant coherence between contralateral motor cortex electrocortical signals and lower limb EMG was observed in the beta- and gamma-range for all exercise types. Gamma-range coherence was significantly greater for isotonic exercises than for isometric exercises. We conclude that active muscle movement modulates the speed of corticospinal oscillations. Specifically, isotonic contractions shift corticospinal oscillations toward the gamma-range while isometric contractions favor beta-range oscillations. Prior research has suggested that tasks requiring increased integration of visual and somatosensory information may shift corticomuscular coherence to the gamma-range. The isometric and isotonic tasks studied here likely required similar amounts of visual and somatosensory integration. This suggests that muscle dynamics, including the amount and type of proprioception, may play a role in the beta-to-gamma shift.

## Introduction

Coherence between electroencephalography (EEG) recorded on the scalp above the motor cortex and electromyography (EMG) recorded on the skin over muscles is thought to reflect corticospinal coupling between motor cortex and pooled motor units (Mima and Hallett, 1999; Negro and Farina, 2011). Corticomuscular coherence phase lags are consistent with the conduction time between the motor cortex and the respective muscle. This suggests that the motor cortex drives the motorneuron pool (Gross et al., 2000).

Studies of corticomuscular (EEG-EMG) coherence have largely focused on the upper limbs (Halliday et al., 1998; Mima et al., 2000; Kristeva-Feige et al., 2002; Kristeva et al., 2007; Omlor et al., 2007; Chakarov et al., 2009; Yang et al., 2009). The prevalence of monosynaptic corticospinal projections to the motor units of the upper limbs, and the hand in particular, contributes to the dexterity of the upper limbs compared to the lower limbs (Krakauer and Ghez, 2000). There have been a few studies investigating EEG-EMG coherence for lower limbs muscles (Mima et al., 2000; Hansen et al., 2002; Raethjen et al., 2008; Vecchio et al., 2008). For both the upper and lower limbs, the existence of these causal descending signals reflects motor cortex control of voluntary movements via pyramidal pathways (Mima and Hallett, 1999; Gross et al., 2000; Negro and Farina, 2011).

The type of motor task affects the frequency band where corticomuscular coherence is most prominent. Beta-range (13–30 Hz) corticomuscular coherence measured using EEG has been extensively documented during static force output (Gross et al., 2000; Mima et al., 2000; Kristeva-Feige et al., 2002; Kristeva et al., 2007; Raethjen et al., 2008; Chakarov et al., 2009; Yang et al., 2009). Gamma-range (31–45 Hz) corticomuscular coherence has been studied to a lesser extent but was first reported during strong contraction (Brown et al., 1998; Mima and Hallett, 1999) and has recently been linked to dynamic force output (Marsden et al., 2000; Omlor et al., 2007). Marsden et al. recorded electrocorticographic (ECoG) signals from non-pathological areas in humans with subdural electrodes in place for investigation of epilepsy. There was coherence between ECoG and simultaneously recorded EMG from upper limb muscles in the beta-range for isometric contractions and in the gamma-range for self-paced phasic contractions. Omlor et al. evaluated EEG-EMG coherence during constant and periodically modulated force production in a visuomotor task (i.e., tracking a sinusoidal force given visual force feedback). In both tasks, subjects attempted to achieve a target force given real-time visual feedback of force production. For the constant force condition, EEG-EMG coherence existed in the beta-range. For the periodically modulated force condition, the EEG-EMG coherence shifted toward higher (gamma-range) frequencies.

A complete explanation for the beta-to-gamma corticomuscular coherence shift for static versus dynamic tasks is lacking. Omlor et al. hypothesized that the shift toward higher frequencies for the dynamic force tracking task compared to the constant force task reflected that tracking a periodically modulated force requires more attentional resources and more complex integration of visual and somatosensory information than the constant force task. They suggested that higher frequency coherence might reflect the integration of multi-sensory information into the motor plan. However, Marsden et al. observed a beta-to-gamma shift for a self-timed task without visual feedback.

The purpose of the present study was to compare corticomuscular coherence for isometric and isotonic contractions when both contraction types were self-paced and in the absence of external force feedback. We hypothesized that despite similar visual and sensory motor integration demands for both tasks the isotonic contractions would elicit gamma-range corticomuscular coherence while the isometric contractions would elicit beta-range coherence. We based this hypothesis on the observation from Marsden et al. that self-paced phasic contractions shifted corticomuscular coherence to the gamma-range in the absence of visuomotor coordination. A novel aspect of our study is that we used independent components analysis (Makeig et al., 1996; Jung et al., 2000a; Onton et al., 2006; Delorme et al., 2012) to separate out motor cortex electrocortical sources rather than directly using EEG electrode signals for calculating corticomuscular coherence.

## Methods

### Data collection

The experimental apparatus, testing protocol, and data collection procedures have been described previously (Gwin and Ferris, 2012) and are briefly summarized here. The subjects of this study were eight healthy right-handed and right-footed volunteers with no history of major lower limb injury and no known neurological or musculoskeletal deficits (seven males; one female; age range 21–31 years). These subjects sat on a bench while performing isometric muscle activations and isotonic movements (concentric followed by eccentric) of the right knee and right ankle joints. Exercise repetitions took approximately 3 s. For isotonic tasks concentric and eccentric contractions were performed continuously (i.e., immediate direction change after the concentric contraction). Subjects paused for 5 s between repetitions. We did not provide timing cues because we did not want to confound electrocortical dynamics with an audio or visual task. As a result, exercise timing was approximate.

We recorded EEG using an ActiveTwo amplifier with a 512 Hz low-pass filter and a 264-channel active electrode array (BioSemi, Amsterdam, The Netherlands). We recorded lower limb EMG at 1000 Hz (tibialis anterior, soleus, vastus lateralis, vastus medialus, medial gastrocnemius, lateral gastrocnemius, medial hamstring, and rectus femoris) using eight surface EMG sensors and a K800 amplifier (Biometrics, Gwent, England), as well as a Vicon data acquisition system (Vicon, Los Angeles, US). The University of Michigan Internal Review Board approved all procedures, which complied with the standards defined in the Declaration of Helsinki.

### EEG and EMG pre-processing

EEG was pre-processed in the same manner as (Gwin and Ferris, 2012) using Matlab (The Mathworks, Natick, MA) scripts based on EEGLAB, an open source environment for processing electrophysiological data (Delorme and Makeig, 2004). We applied a zero phase lag 1 Hz high-pass Butterworth filter to the EEG signals to remove drift. Next, we removed EEG signals exhibiting substantial noise throughout the collection; the channel rejection criteria were standard deviation greater than 1000 μV, kurtosis more than three standard deviations from the mean of all channels, or correlation coefficient with nearby channels less than 0.4 for more than 0.1% of the time-samples. The remaining channels were average referenced (191 ± 34.6 channels, mean ± standard deviation). For each subject, we submitted these channel signals to a 2-model adaptive mixture independent component analysis (AMICA) (Palmer et al., 2006, 2008; Delorme et al., 2012). We have previously demonstrated that applying a 2-model AMICA decomposition to these data captures differences in the electrocortical source distribution for knee versus ankle exercises (Gwin and Ferris, 2011, 2012). DIPFIT functions (Oostenveld and Oostendorp, 2002) within EEGLAB computed an equivalent current dipole model that best explained the scalp topography of each independent component using a boundary element head model based on the Montreal Neurological Institute (MNI) template. We excluded independent components if the projection of the equivalent current dipole to the scalp accounted for less than 85% of the scalp map variance, or if the topography, time-course, and spectra of the independent component were reflective of eye movement or electromyographic artifact (Jung et al., 2000a,b). The remaining independent components reflected electrocortical sources. EEGLAB clustered electrocortical sources across subjects based the equivalent current dipole models of the sources. We retained clusters that contained electrocortical sources from at least six of eight subjects; the geometric means of these clusters were in contralateral motor (two clusters), ipsilateral motor, anterior cingulate, posterior cingulate, and parietal cortex (Gwin and Ferris, 2011, 2012). Electrocortical sources that were not included in these clusters were excluded from all further analyses. EMG signals were re-sampled at 512 Hz (the EEG sampling rate) using the Matlab *resample* function and then full-wave rectified. Full wave rectified surface EMG mimics the temporal pattern of grouped firing motor units (Halliday et al., 1995). The onset and offset of each exercise repetition were determined based on the onset and offset of applied force (Omegadyne load cell, Sunbury, OH, USA) for isometric exercises and joint rotation (Biometrics electrogoniometer, Gwent, England) for isotonic exercises.

### Corticomuscular coherence

For each exercise set (i.e., 20 repetitions) the power spectra of rectified EMG, EEG, and electrocortical source signals were computed using Welch's method with 0.5 s non-overlapping Hanning windows (for a frequency resolution of 2 Hz). Only active data (i.e., between onset and offset of each exercise repetition) were used for power spectral estimation. Magnitude squared coherence was computed as follows for each EEG channel/EMG channel pair and for each electrocortical source/EMG channel pair:

(1) $${\text{coh}}_{c1,c2}\left(f\right)=\frac{{\left|S_{c1c2}\left(f\right)\right|}^{2}}{S_{c1c1}\left(f\right)\cdot S_{c2c2}\left(f\right)}$$

where *S*_*c*1*c*1_ and *S*_*c*2*c*2_ are the auto-spectra of each signal; and *S*_*c*1*c*2_ is the cross-spectra. Coherence was only computed for agonist muscles (i.e., for flexion exercises coherence was computed for tibialis anterior and medial hamstring, and for extension exercises coherence was computed for soleus, medial gastrocnemius, lateral gastrocnemius, vastus lateralis, vastus medialus, and rectus femorus). Coherence was considered to be significant if it was greater than the 95% confidence limit (CL), which was computed as follows (Rosenberg et al., 1989):

(2) $$\text{CL}=1-0.05^{\frac{1}{n-1}}$$

where *n* is the number of windows used for spectral estimation. In this study the number of windows was not the same for all spectral estimates because exercises were self-paced. Therefore, coherence values were linearly warped so that the 95% CL was the same for all coherence estimates.

Coherence scalp-maps visualizing the maximum EEG-EMG coherence in the alpha-range (8–12 Hz), beta-range (13–30 Hz), and gamma-range (31–45 Hz) for each EEG channel/EMG channel pair were computed for each subject and exercise set. Grand average coherence scalp-maps were generated for isometric and isotonic exercises by first interpolating subject specific coherence maps to a standardized 64-channel electrode array (using spherical interpolation implemented in EEGLAB) and then averaging interpolated coherence maps across subjects. Interpolation to a standardized 64-channel electrode array was necessary because after EEG-channel rejection the electrode montages were not consistent across subjects.

Peak coherence in the beta- and gamma-range was computed for each electrocortical source/EMG channel pair. Finding significant coherence only for the contralateral motor cortex cluster, a two-way analysis of variance (ANOVA) was used to assess the significance of differences in grand average coherence peaks for independent variables frequency (beta versus gamma) and exercise type (isometric versus isotonic). The significance criteria was set at α = 0.05 *a priori* and Bonferroni correction was used to address the problem of multiple comparisons.

## Results

**Figure 1:**
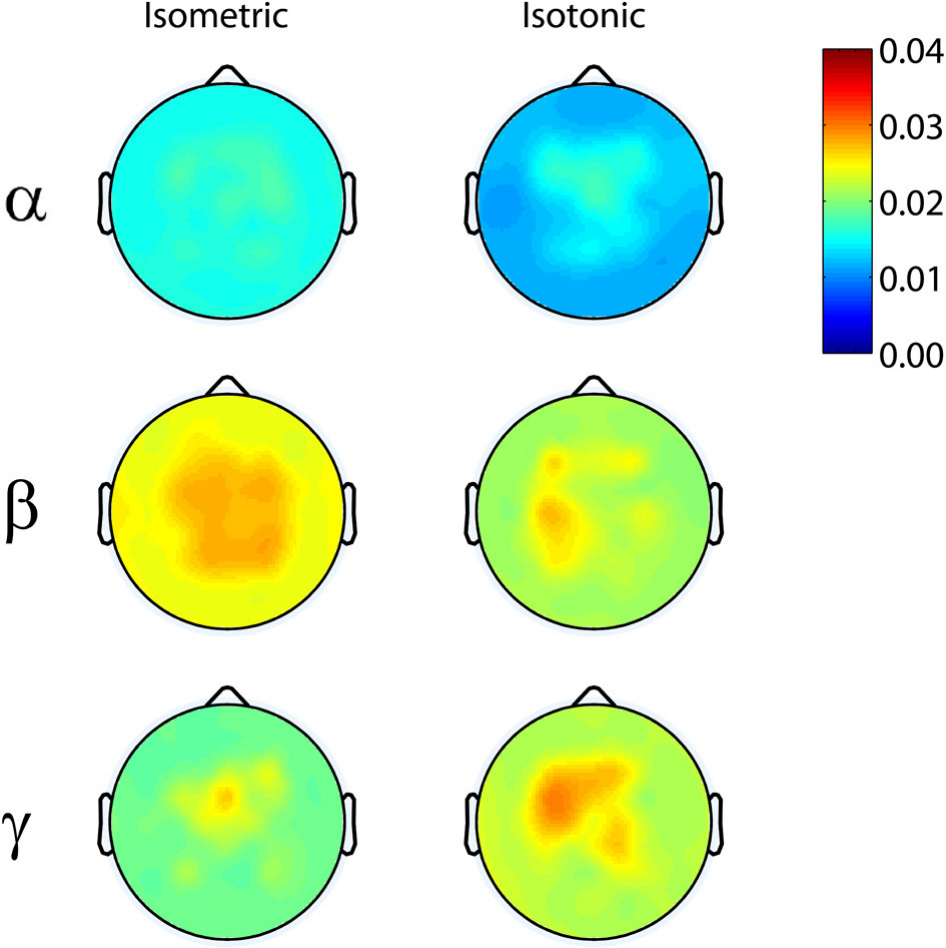
**Grand average (top row) alpha-range, (middle row) beta-range, and (bottom row) gamma-range EEG-EMG coherence scalp-maps for (left) isometric and right (isotonic) exercises.** 95% coherence confidence limit = 0.025.

**Figure 2:**
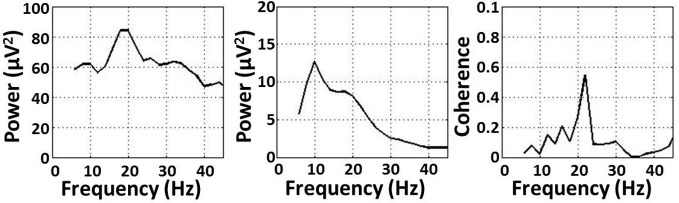
**(Left) EMG power, (middle) EEG power, and (right) coherence for a representative subject performing an isometric exercise**.

Significant coherence between EEG-channel signals and lower limb EMG was observed in the beta- and gamma-range, but not in the alpha-range, for all exercise types (Figure 1). The coherence peaks occurred at 22.3 ± 5.1 Hz and 37.9 ± 4.2 Hz for the beta and gamma bands, respectively. These frequency peaks are not harmonics of each other. Peaks in the EMG spectral power occurred at 17.3 ± 3.9 Hz and 35.1 ± 3.7 Hz for the beta and gamma bands, respectively. EEG spectral power, EMG spectral power, and coherence are shown for a representative subject performing an isometric exercise (Figure 2). Beta-range coherence for isometric exercises was broadly and bilaterally distributed over the medial sensorimotor cortex and favored the contralateral side (left column, middle row, Figure 1). Beta-range coherence for isotonic exercises was distributed less broadly and was only significant only over the contralateral sensorimotor cortex (right column, middle row, Figure 1). Gamma-range coherence for isometric exercises was distributed narrowly over the medial motor cortex (left column, bottom row, Figure 1). Gamma-range coherence for isotonic exercises was distribute more broadly and favored the contralateral side (right column, bottom row, Figure 1).

**Figure 3:**
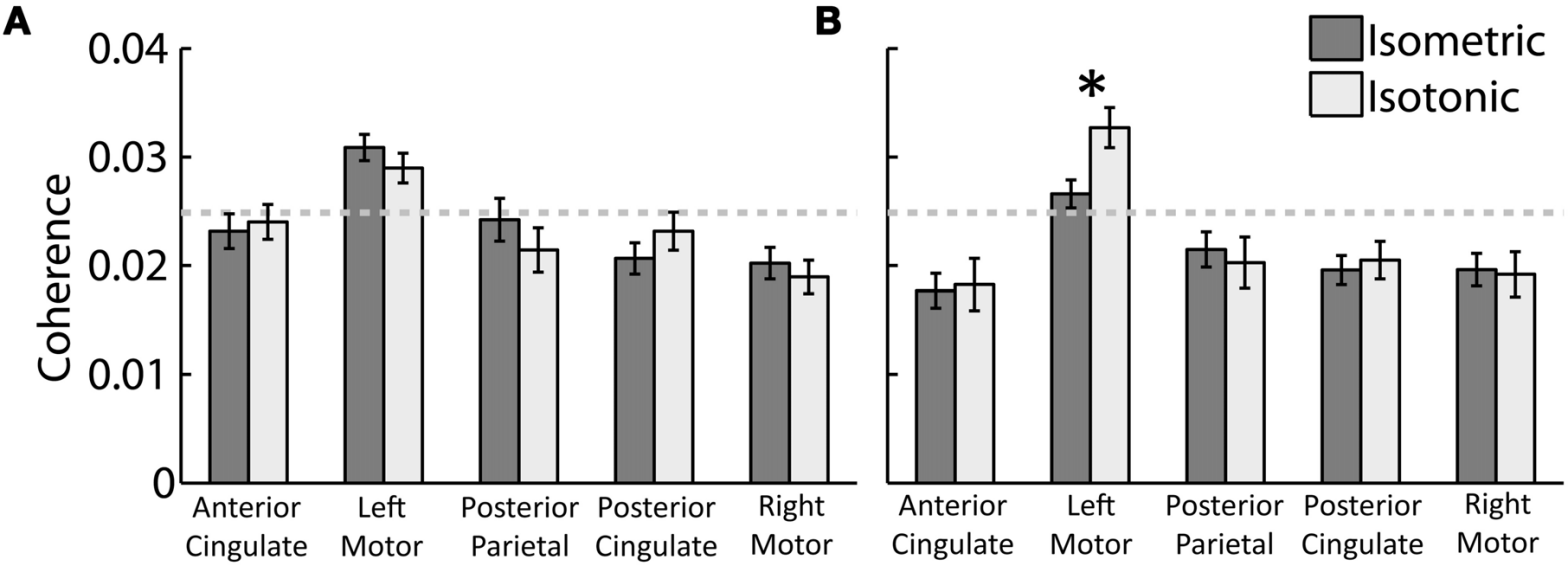
**Grand average peak (A) beta-range and (B) gamma-range coherence between EMG and electrocortical source signals for (dark grey) isometric and (light grey) isotonic exercises.** The 95% coherence confidence limit is indicated with a dashed grey line. Error bars show the standard error of the mean. ^*^Indicates a significant difference between isometric and isotonic conditions (*p* < 0.05).

**Figure 4:**
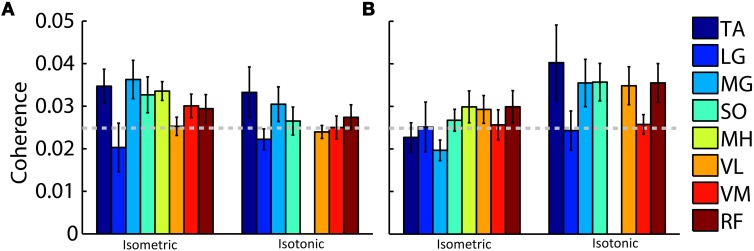
**Grand average peak (A) beta-range and (B) gamma-range coherence between contralateral motor cortex electrocortical source signals and EMG for isometric and isotonic exercises.** Colored bars represent (TA) tibialis anterior, (LG) lateral gastrocnemius, (MG) medial gastrocnemius, (SO) soleus, (MH) medial hamstring, (VL) vastus lateralis, (VM) vastus medialus, and (RF) rectus femoris muscles. The 95% coherence confidence limit is indicated with a dashed grey line. Error bars show the standard error of the mean. Isotonic knee flexion could not be accommodated by the test apparatus; therefore, no values are shown for isotonic MH coherence.

Beta- and gamma-range coherence between contralateral motor cortex electrocortical source signals and lower-limb EMG was significant for all exercise types (Figure 3). A two-way ANOVA with independent variables exercise type (isometric versus isotonic) and frequency band (beta versus gamma) did not show any significant main effects. However the interaction between independent variables was significant (*p* < 0.05). Further assessments of the marginal means (using Bonferroni correction) demonstrated that in the gamma-range, coherence for isotonic exercises was significantly greater (*p* < 0.05) than coherence for isometric exercises. These coherence values are separated by muscle in Figure 4. The trend of increased gamma-range coherence for isotonic compared to isometric exercise was consistent across all muscles except vastus medialus and lateral gastrocnemius, which did not exhibit significant coherence for either condition. Anterior cingulate, posterior cingulate, posterior parietal, and ipsilateral motor electrocortical source signals did not exhibit significant coherence with EMG.

## Discussion

We found that both isometric and isotonic, knee and ankle exercises elicited small but significant coherence between contralateral motor cortex electrocortical signals and lower limb EMG in the beta- and gamma-range. We hypothesized that isotonic contractions would elicit gamma-range corticomuscular coherence while the isometric contractions would elicit beta-range coherence. What we found was that both tasks elicited beta and gamma range coherence. Beta-range coherence was slightly but not significantly greater for isometric tasks than for isotonic tasks and gamma-range coherence was significantly greater for isotonic exercises than for isometric exercises. This finding is consistent with prior research using ECoG to study corticomuscular coherence during tonic and phasic contractions (Marsden et al., 2000) and suggests that muscle dynamics and relative changes in proprioception may play a role in the beta-to-gamma shift of coherent frequencies for static versus dynamic force production.

Gamma-range corticomuscular coherence has also been observed using scalp EEG during an isometric force tracking task when subjects attempted to achieve a periodically modulated target force given real-time visual feedback of force production (Omlor et al., 2007). The authors of that study hypothesized that the shift toward higher (gamma-range) frequencies might have reflected the fact that tracking a periodically modulated force requires more attentional resources and more complex integration of visual and somatosensory information for control than tracking a constant force. We observed a similar beta-to-gamma shift but the isotonic task studied here did not require more visuomotor integration than the isometric task. Despite the fact that the external anatomy remains stationary, isometric force increases involve dynamic muscle shortening and tendon lengthening while isometric force decreases involve muscle lengthening and tendon shortening (Fukunaga et al., 2002). Therefore, the beta-to-gamma shift observed here may not be inconsistent with the beta-to-gamma shift for the periodically modulated isometric force production task used by Omlor et al. (2007).

Our findings of corticomuscular coherence were consistent across most of the muscles of the lower limb. We recorded EMG from tibialis anterior, soleus, vastus lateralis, vastus medialus, medial gastrocnemius, lateral gastrocnemius, medial hamstring, and rectus femoris muscles. We found that the beta-to-gamma coherence frequency shift was consistent across all muscles (i.e., isotonic contractions elicited greater gamma-range coherence than isometric contractions) except vastus medialus and lateral gastrocnemius, which did not exhibit significant coherence for either condition. This observation is consistent with a common pyramidal pathway activating multiple coordinated muscles via spinal interneurons to achieve coordinated limb movement at a lower computational cost (Krakauer and Ghez, 2000; Ting and McKay, 2007).

Most EEG-based studies of corticomuscular coherence evaluate coherence between scalp EEG and surface EMG signals. However, many underlying source signals (including electrocortical, electroocular, electromyographic, and artifact sources) collectively contribute via volume conduction to the electrical potentials recorded on the scalp. These sources can be parsed from scalp EEG using blind source separation techniques and equivalent current dipole modeling (Delorme et al., 2012). In this study, multi-subject clusters of electrocortical sources were localized to the contralateral motor (two clusters), ipsilateral motor, anterior cingulate, posterior cingulate, and parietal cortex. However, only the electrocortical sources in the contralateral motor cortex exhibited significant corticomuscular coherence. This finding is consistent with the knowledge that the corticospinal pathways originate in the motor cortex. Interestingly, the magnitudes of the source-to-EMG correlation and the scalp-to-EMG correlations were not substantially different. In addition, we evaluated scalp-to-EMG correlations after removing non-brain independent components and did not see a significant difference in the correlation scalp topography. Nevertheless, the use of independent components analysis to separate out motor cortex sources rather than directly using EEG electrode signals for calculating corticomuscular coherence is beneficial because it ensures that mixing of various electrocortical processes, as well as neck and facial EMG signals, via volume conduction doesn't bias the analysis. In this study we used a standardized head model to localize electrocortical sources. Future work should examine the use of subject specific head models based on magnetic resonance images for each subject. This technique, which is available in EEGLAB, can improve the localization accuracy. Blind source separation techniques like AMICA, combined with subject specific head models, may be beneficial for future studies of corticomuscular coherence, particularly during dynamic motor tasks when scalp EEG signals can be highly contaminated by electromyographic and movement artifacts (Gwin et al., 2010a,b; Gramann et al., 2011).

In conclusion, significant coherence between contralateral motor cortex electrocortical signals and lower limb EMG was observed in the beta- and gamma-range for both isometric and isotonic self-paced knee and ankle exercises. However, gamma-range coherence was significantly greater for isotonic exercises than for isometric exercises. This beta-to-gamma shift was consistent across six of the eight lower limb muscle EMG signals that we recorded. This suggests that active muscle movement may modulate the speed of corticospinal oscillations. Specifically, isotonic contractions shift corticospinal oscillations towards the gamma-range while isometric contractions favor beta-range oscillations.

### Conflict of interest statement

The authors declare that the research was conducted in the absence of any commercial or financial relationships that could be construed as a potential conflict of interest.
